# Nanodisc-T_m_: Rapid functional assessment of nanodisc reconstituted membrane proteins by CPM assay

**DOI:** 10.1016/j.mex.2016.03.009

**Published:** 2016-03-14

**Authors:** Yashwanth Ashok, Veli-Pekka Jaakola

**Affiliations:** Faculty of Biochemistry and Molecular Medicine & Biocenter Oulu, University of Oulu, Oulu 90014, Finland

**Keywords:** Nanodisc-T_m_, Membrane proteins, Nanodiscs, CPM assay

## Abstract

Membrane proteins are generally unstable in detergents. Therefore, biochemical and biophysical studies of membrane proteins in lipidic environments provides a near native-like environment suitable for membrane proteins. However, manipulation of proteins embedded in lipid bilayer has remained difficult. Methods such as nanodiscs and lipid cubic phase have been developed for easy manipulation of membrane proteins and have yielded significant insights into membrane proteins. Traditionally functional reconstitution of receptors in nanodiscs has been studied with radioligands. We present a simple and faster method for studying the functionality of reconstituted membrane proteins for routine characterization of protein batches after initial optimization of suitable conditions using radioligands. The benefits of the method are

• Faster and generic method to assess functional reconstitution of membrane proteins.
• Adaptable in high throughput format (≥96 well format).
• Stability measurement in near-native lipid environment and lipid dependent melting temperatures.

## Graphical abstract

**Figure:**
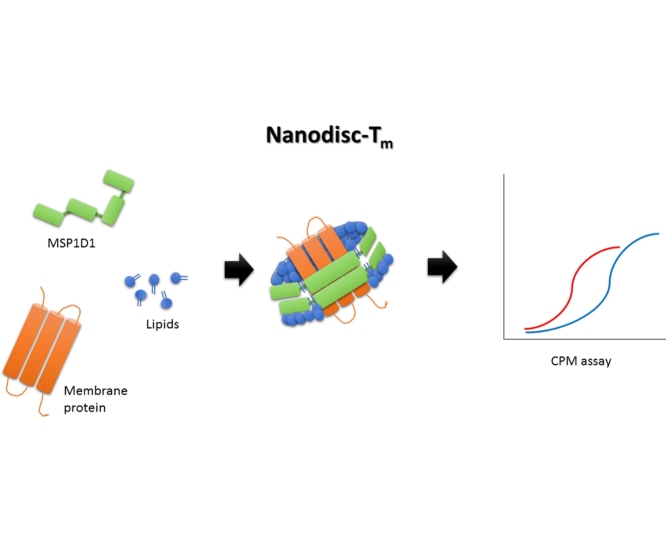

## Method details

### Adenosine A2A receptor purification

Prepare 2% (w/v) cholesteryl hemi succinate (CHS) dissolved in 10% (w/v) *n*-dodecyl β-d-maltoside (DDM). Expression cassette for human adenosine A_2A_ receptor (A_2A_R) consisted of hemagglutinin signal sequence, FLAG epitope, 10X histidine (His) tag, T4 lysozyme and amino acids encoding the receptor from 1 to 317 residues in pFastBac1 vector using *Spodoptera frugiperda* (Sf9) insect cells. Cell membranes were expressed and washed as described previously [3], [9]. Prior to solubilisation thaw the membranes on ice with 4 mM theophylline.

1. Add iodoacetamide to a concentration of 2 mg/ml and incubate at 4 °C for 30 min. Membranes were solubilized by adding 2× solubilization buffer (50 mM Hepes pH 7.5, 1600 mM NaCl, 20% glycerol, 2% DDM-CHS mixture and 4 mM theophylline and incubated at 4 °C for 2.5 h. Unsolubilized membranes were centrifuged at 150,000*g* for 45 min.
2. For 1 l of biomass 1 ml of TALON (Clontech) immobilized metal affinity chromatography (IMAC) resin was used. The resin was pre-equilibrated with solubilization buffer. The supernatant was incubated with the resin overnight at 4 °C with 20 mM imidazole.
3. After overnight binding, the resin was washed with 10 column volume (10 ml) of wash buffer I (250 mM Hepes pH 7.4, 800 mM NaCl, 10 mM MgCl_2,_ 0.1% DDM:CHS, glycerol 10%, 25 mM imidazole, 8 mM adenosine triphosphate (ATP) and 100 μM ZM341285. This was followed by wash buffer II (50 mM Hepes pH 7.4, 800 mM NaCl, and 0.05% DDM-CHS, 10% glycerol, 50 mM imidazole and 100 μM ZM341285). The receptor was eluted in 25 mM Hepes pH 7.4, 800 mM NaCl, and 0.01% DDM: CHS, 10% glycerol, 220 mM imidazole and 100 μM ZM341285.
4. Concentrate the eluted receptors in a 100 kDa centricon centrifugal filter. Assess purity and monodispersity by sodium dodecyl sulphate-polyacrylamide gel electrophoresis (SDS-PAGE) and analytical size-exclusion chromatography (aSEC).

## Assembly of nanodiscs

1-Palmitoyl-2-oleoyl-*sn*-glycero-3-phosphocholine (POPC) and 1-palmitoyl-2-oleoyl-*sn*-glycero-3-phospho-l-serine (POPS) were purchased from Avanti Polar Lipids. We have used Bio-beads, Biorad and Amberlite XAD-2, Sigma–Aldrich with equal success.

1. Solubilize lipids using sodium cholate detergent. Cholate concentration must be twice as lipid concentration. For example, solubilize 100 mM lipids in 200 mM cholate.
2. Using PD-10 column buffer exchange the receptor to 50 mM Hepes pH 7.4, 800 mM NaCl and 0.01% DDM-CHS.
3. Mix A_2A_R: Membrane Scaffold Protein 1D1 (MSP1D1): lipid in a ratio of 1:10:700. POPC:POPS (7:3) was used in reconstitution. POPS is a negatively charged lipid. This is added to mimic the negative charge of the inner leaflet of the plasma membrane. The above mentioned ratio has been optimized to yield monomeric adenosine A_2A_ receptor in nanodiscs. Use His tag cleaved MSP1D1 protein.
4. Incubate the mixture with wet bio beads (170 mg of beads for 250 μl reaction) overnight at 4 °C to capture detergents and to aid in nanodiscs formation. Next day puncture a hole in the bottom of the tube and place it in a 15 ml falcon tube. Centrifuge the falcon tube to collect the mixture.
5. Pre-equilibrate IMAC resin with 50 mM Hepes pH 7.4, 150 mM NaCl and 25 mM imidazole. Use 200 μl Ni-NTA (nickel-nitrilotriacetatic acid) agarose column volume. His tag is present only in receptors and MSP1D1 does not have any His tag. Thus empty nanodiscs are removed in this step. Wash with 10 column volumes of 50 mM Hepes pH 7.4, 150 mM NaCl and 50 mM imidazole. Elute the bound proteins with 50 mM Hepes pH 7.4, 150 mM NaCl and 500 mM imidazole. This IMAC procedure effectively removes empty nanodiscs and purifies only nanodisc reconstituted receptors.
6. Concentrate the eluted A_2A_ receptor-nanodisc with a 30 kDa cut-off concentrator while simultaneously exchanging the buffer to 50 mM Hepes pH 7.4, 150 mM NaCl.
7. As the above mentioned ratio is optimized for GPCR (G protein-coupled receptors) incorporation into nanodiscs, we rarely find any aggregation. Thus, we only run an analytical scale size exclusion chromatography for verification.
8. Check reconstitution by SDS-PAGE and radioligand binding (if applicable).

## Analytical size exclusion chromatography (aSEC)

Size exclusion chromatrography (SEC) buffer typically contained 50 mM Hepes pH 7.5, 150 mM NaCl. aSEC was performed in Shimadzu High performance liquid chromatography (HPLC) system with autoinjection device coupled to ultra violet (UV) detector. SEC was performed in Sepax Nanofilm-250 (column dimensions 4.6 × 300 mm) (Sepax Technologies, Inc.) with a flowrate of 500 μl/min and injection volume of 20 μl.

## CPM assay

MSP1D1 protein has no cysteines in its sequence, whereas the receptor used in reconstitution has cysteines. CPM is a cysteine specific fluorophore and thus any fluorescence signal emitted from the sample is specific to the receptor.

1. Dissolve *N*-[4-(7-diethylamino-4-methyl-3-coumarinyl)phenyl] maleimide (CPM) dye in dimethyl sulfoxide (DMSO) at a concentration of 4 mg/ml and store in −80 °C. CPM assay was essentially conducted as described in [2].
2. Prior to use the dye was diluted 1:40 in CPM dilution buffer 20 mM Hepes pH 7.5, 150 mM NaCl for nanodisc samples, whereas for detergent micelle containing samples the same buffer with 0.05% DDM was used. The reaction was performed in a total of 120 μl. 1–5 μg of the protein was diluted in CPM dilution buffer in the case of nanodisc samples and supplemented with 0.05% DDM for micellar samples. Ligands if required were aded to 110 μl and 10 μl of diluted dye was added and incubated at room temperature for 10 min in dark. Ligands were used at a concentration of 100 μM. The mixture was transferred to a micro quartz cuvette and heated in a controlled manner (2 °C/min) in a fluoromax-4 (Horiba scientific) fluorometer. The excitation was at 387 nm and emission was recorded in 463 nm
3. The temperature recorded was from 20 to 100 °C. Data analysis was performed in Graph pad with Boltzmann sigmoidal fitting. Data was normalized against initial fluorescence values.

## Method validation

**Fig. 1:**
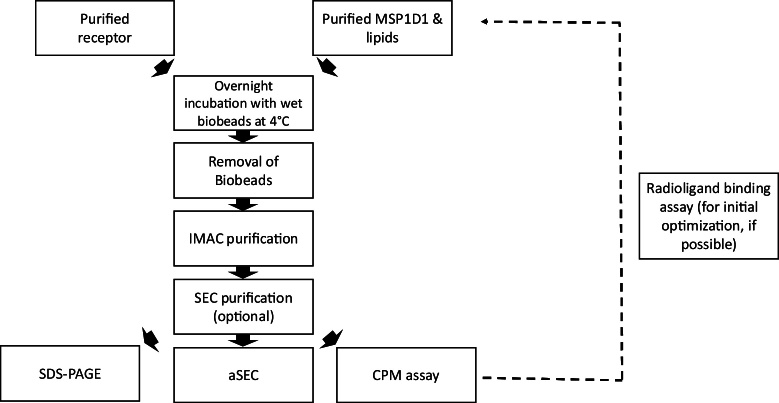
Flow diagram of methods used for nanodisc reconstitution.

An overview of the reconstitution procedure is shown in Fig. 1. Numerous reports have been published for optimal protocol for reconstitution of GPCRs and validated using radioligands [10], [12], [16]. Here we focus on the use of CPM assay for determining functionality of receptors in nanodiscs and for assessing stability of membrane proteins in a lipidic environment. CPM assay is routinely used for studying the stability of membrane proteins [2]. CPM is a cysteine specific binding dye. Membrane proteins have cysteine residues in transmembrane regions which are exposed upon unfolding. These newly exposed cysteines bind to CPM and emits fluorescence. To evaluate the usefulness of CPM assay for nanodisc reconstituted membrane proteins, we used human adenosine A_2A_ receptor reconstituted in nanodiscs as an example. The receptor construct used in this study has a total of fourteen cysteine residues of which eight are involved in formation of disulphide bonds. Six cysteine residues are free to react with CPM dye.

**Fig. 2:**
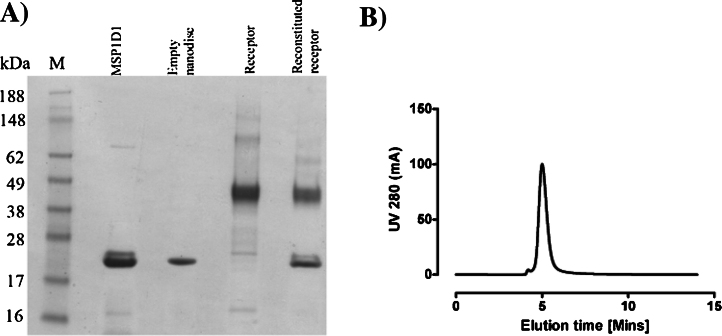
(A) SDS-PAGE analysis of reconstituted receptor in nanodisc. Reconstituted receptor has both bands corresponding to the receptor and MSP1D1. Empty nanodisc samples have only MSP1D1 protein. (B) aSEC analysis of nanodisc reconstituted receptors. Samples were run without any ligands.

A lipid mixture of POPC:POPS was used to mimic zwitterionic environment of the plasma membrane. Fig. 2A shows an example of successful reconstitution of the receptor into the discs. Fig. 2B shows aSEC profile of the IMAC purified mixture indicating the sample is monodisperse.

**Fig. 3:**
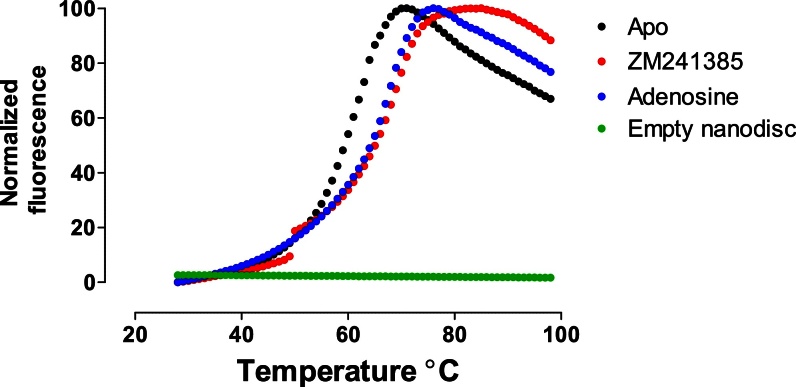
CPM analysis of nanodisc reconstituted receptors. Dotted objects circles indicate the CPM signals: for A_2A_ in nanodiscs alone (black) (T_m_ = 58 °C), with ZM241385 (red) (T_m_ = 63 °C) and adenosine (blue) (T_m_ = 64 °C). Empty nanodisc samples are indicated with green circles.

Membrane scaffold proteins (MSP) lack cysteines and lipids commonly used in reconstitution do not have any sulfhydryl groups that would bind to CPM dye. Empty nanodiscs (nanodiscs without any incorporated membrane proteins) were used as a control. Fig. 3 shows that there was no fluorescence signal with empty nanodisc samples, whereas adenosine receptor incorporated samples showed a sigmoidal transition which is typical for CPM assay. We then investigated if ligands for adenosine A_2A_ receptor could stabilize the receptor when compared to unliganded (apo) state. We chose ZM241385, a high affinity sub-type specific binding antagonist and adenosine which is the natural ligand for the receptor. Both the ligands enhanced the stability of the receptor. Apo state had a melting temperature (T_m_) of 58 °C and adenosine bound samples increased the melting temperature by 5 °C (T_m_ = 63 °C). ZM241385 on the other hand increased the stability of the receptor by 6 °C (T_m_ = 64 °C), more than adenosine. This is expected as ZM241385 is a much better ligand for the receptor in terms of affinity. Adenosine has a half maximal effective concentration (EC_50_) of roughly 1 μM and ZM241385 has an equilibrium dissociation constant (K_D_) of 2.1 nM [6], [9]. Given this huge difference in affinity, one might expect more than 1 °C stabilization for ZM241385. We do not observe a huge difference in stability between the ligands as the receptor to start with is already in a stable environment of lipid bilayer and the assay is no longer linear. This has been observed before during thermostabilization of human adenosine A_2A_ receptor in agonist bound conformation. Thermostabilization of single, double and triple mutants was done in a mild detergent (DDM) but to further find additive thermostabilizing mutations, the assay had to be done in decyl maltoside (DM) which has a shorter detergent chain length for quadruple mutants [11].

Stabilization effect of the sample is a direct consequence of ligand binding and therefore indicating that the reconstituted sample is active in ligand binding. Since the CPM signal responds to the ligands, it is certain that the signal acquired in the assay belongs to adenosine receptor.

Similar experiments were conducted with purified receptor in DDM-CHS micelles for comparing stabilization effect of lipids used reconstitution mixture. Table 1 shows the melting temperature of receptor in micelles and nanodiscs. Clearly nanodisc reconstituted samples are more stable than micelle samples. Lipids used in reconstitution of nanodiscs stabilize the receptor by 14 °C with an apparent T_m_ of 44 °C for DDM-CHS purified apo receptor. Adenosine bound receptors in micelles had a T_m_ of 57 °C. ZM241385 bound receptors had an apparent T_m_ of 59 °C.

In summary, our results show that nanodisc reconstituted membrane proteins can be used to study stability of membrane proteins using CPM assay.

## Additional information

Membrane proteins are extracted from the membranes using detergents. As detergents are poor mimics of lipids, membrane proteins are generally unstable in them. This property has rendered biochemical studies of membrane proteins difficult. Lipid cubic phase has been used as a membrane mimic for studying catalysis of an integral membrane enzyme [13]. Nanodiscs which are based on apolipoprotein A-I have been used to reconstitute membrane proteins in lipid bilayer-like environment [4]. Insertion of membrane protein of interest into nanodiscs allows easy manipulation of the proteins in a similar manner to soluble proteins. This is particularly advantageous as the experiments are conducted in a minimal detergent environment. In addition, membrane proteins are much more stable as they are inserted into a lipid bilayer. Nanodisc reconstituted membrane proteins have been used in a wide range of studies from protein–protein interactions to single molecule fluorescence spectroscopy [1], [10].

Traditionally reconstituted receptors have been assessed for functionality by using radioligand binding assays. However, many receptors such as olfactory receptors are orphan and therefore do not have any radioligands to assess activity. In these cases one can use only SDS-PAGE and SEC for analysis. Therefore a generic assay independent of membrane protein used in reconstitution is required.

Cherezov and co-workers have used CPM assay successfully in lipid cubic phase [14]. We hypothesized that CPM assay could be used to study nanodisc reconstituted receptors. In cases where radioligand based optimizations are not possible, we believe that CPM assay is an alternative. If radioligand based optimizations are possible, then one can get complimentary information from both CPM and radioligand based assays. Nanodisc-T_m_ assay is applicable to all classes of membrane proteins such as enzymes, ion channels, transporters and receptors.

Lipids have been shown to modulate G protein binding in case of neurotensin receptor (NTS1) [8]. It is well known that cholesterol modulates ligand binding properties of oxytocin and serotonin receptors [7], [15]. Recently, have shown that specific lipids allosterically modulate pharmacological properties of β1-adrenergic receptor [5]. This assay can also be used to study stabilization effects of specific lipids as well. Combining nanodisc technology with CPM assay could be used in unraveling the role of specific lipids in receptor structure-function studies.

## Figures and Tables

**Table 1:** Thermostability of detergent purified and nanodisc reconstituted A_2A_ receptor.

Apparent T_m_ (°C) (n = 3)		
Condition	DDM-CHS	Nanodisc
Apo	44 ± 1	58 ± 1
Adenosine	57 ± 1	63 ± 1
ZM241385	59 ± 1	64 ± 1
